# Measurement of Pulmonary Flow Reserve and Pulmonary Index of Microcirculatory Resistance for Detection of Pulmonary Microvascular Obstruction

**DOI:** 10.1371/journal.pone.0009601

**Published:** 2010-03-09

**Authors:** Rahn Ilsar, Chirapan Chawantanpipat, Kim H. Chan, Timothy A. Dobbins, Richard Waugh, Annemarie Hennessy, David S. Celermajer, Martin K. C. Ng

**Affiliations:** 1 Royal Prince Alfred Hospital, Sydney, New South Wales, Australia; 2 Department of Medicine, University of Sydney, Sydney, New South Wales, Australia; 3 School of Public Health, University of Sydney, Sydney, New South Wales, Australia; 4 University of Western Sydney, Sydney, New South Wales, Australia; University of Las Palmas de Gran Canaria, Spain

## Abstract

**Background:**

The pulmonary microcirculation is the chief regulatory site for resistance in the pulmonary circuit. Despite pulmonary microvascular dysfunction being implicated in the pathogenesis of several pulmonary vascular conditions, there are currently no techniques for the specific assessment of pulmonary microvascular integrity in humans. Peak hyperemic flow assessment using thermodilution-derived mean transit-time (T*_mn_*) facilitate accurate coronary microcirculatory evaluation, but remain unvalidated in the lung circulation. Using a high primate model, we aimed to explore the use of T*_mn_* as a surrogate of pulmonary blood flow for the purpose of measuring the novel indices Pulmonary Flow Reserve *[PFR = (maximum hyperemic)/(basal flow)]* and Pulmonary Index of Microcirculatory Resistance *[PIMR = (maximum hyperemic distal pulmonary artery pressure)×(maximum hyperemic T_mn_)]*. Ultimately, we aimed to investigate the effect of progressive pulmonary microvascular obstruction on PFR and PIMR.

**Methods and Results:**

Temperature- and pressure-sensor guidewires (TPSG) were placed in segmental pulmonary arteries (SPA) of 13 baboons and intravascular temperature measured. T*_mn_* and hemodynamics were recorded at rest and following intra-SPA administration of the vasodilator agents adenosine (10–400 µg/kg/min) and papaverine (3–24 mg). Temperature did not vary with intra-SPA sensor position (0.010±0.009 *v* 0.010±0.009°C; distal *v* proximal; p = 0.1), supporting T*_mn_* use in lung for the purpose of hemodynamic indices derivation. Adenosine (to 200 µg/kg/min) & papaverine (to 24 mg) induced dose-dependent flow augmentations (40±7% & 35±13% T*_mn_* reductions *v* baseline, respectively; p<0.0001). PFR and PIMR were then calculated before and after progressive administration of ceramic microspheres into the SPA. Cumulative microsphere doses progressively reduced PFR (1.41±0.06, 1.26±0.19, 1.17±0.07 & 1.01±0.03; for 0, 10^4^, 10^5^ & 10^6^ microspheres; p = 0.009) and increased PIMR (5.7±0.6, 6.3±1.0, 6.8±0.6 & 7.6±0.6 mmHg.sec; p = 0.0048).

**Conclusions:**

Thermodilution-derived mean transit time can be accurately and reproducibly measured in the pulmonary circulation using TPSG. Mean transit time-derived PFR and PIMR can be assessed using a TPSG and adenosine or papaverine as hyperemic agents. These novel indices detect progressive pulmonary microvascular obstruction and thus have with a potential role for pulmonary microcirculatory assessment in humans.

## Introduction

The pulmonary microvasculature is the principal site regulating resistance, and thus pressure, in the pulmonary circulation[1]. Diseases of the pulmonary microcirculation, such as pulmonary vascular disease (PVD)[2] and chronic thromboemobolic pulmonary hypertension (CTEPH)[3], are characterized by progressive microvascular obstruction[2], [3], presenting with markedly elevated pulmonary artery pressures (PAP)[4], [5] and thus portending poor prognosis[5], [6]. Currently under recognized[5], [7], both PVD and CTEPH rely on pressure-based diagnostic modalities for detection (eg. echocardiography and right heart catheterization[8]), which are inherently insensitive to the initial pulmonary microvascular losses that precede PAP rise. A more direct assessment of pulmonary microcirculatory status may potentially facilitate improved detection of such conditions.

Advances in miniaturized sensor guidewire technology have enabled the use of both Doppler flow velocity[9] and thermodilution-derived mean transit-time (T*_mn_*)[10] for assessment of the coronary circulation in ischemic heart disease. The index of coronary flow reserve (*CFR  =  maximum hyperemic divided by basal coronary blood flow)* aims to evaluate coronary lesion hemodynamic significance by recruiting all available vascular reserves and expressing maximum resultant flow as a multiple of basal flow. Although capable of detecting microvascular obstruction[11], CFR is not specific to the coronary microcirculation, denoting the combined extent of both epicardial and coronary micrvascular disease in the territory assessed[12]. The recently described index of microcirculatory resistance[13] *(IMR  =  maximum hyperemic distal coronary artery pressure times maximum hyperemic T_mn_)* has been proven to be more microcirculation-specific[14], reproducible[15] and independent of systemic hemodynamics[15] than CFR, tracking true microcirculatory resistance[13]. In contrast, there are currently no established techniques for evaluating the pulmonary microcirculation *in vivo,* a unique vascular bed characterized by low arterial pressure, high vessel compliance and close proximity to alveolar air.

We previously described the use of Doppler flow velocity in hemodynamic assessment of the pulmonary circulation[16] and thus utilized this technology to recently validate and measure pulmonary flow reserve *(PFR  =  maximum hyperemic divided by basal pulmonary blood flow)* in healthy baboons[17]. The use of temperature and pressure sensor-guidewire (TPSG) technology (which facilitates simultaneous thermodilution-derived flow and pressure recordings thus enabling concurrent CFR and IMR evaluations in the coronary circulation[15]), however, has not been described in the pulmonary circulation. We hypothesized that: 1) PFR and a novel pulmonary index of microcirculatory resistance *(PIMR  =  maximum hyperemic distal PAP times maximum hyperemic mean transit-time)* could be measured accurately using a TPSG and; 2) that these novel flow and resistance indices may allow the detection of microvascular obstruction. We thus utilized a high-primate model of invasive pulmonary hemodynamic assessment[17] in order to: 1) demonstrate the feasibility of thermodilution-derived mean transit-time (T*_mn_*)-based pulmonary blood flow assessments using a TPSG and; 2) demonstrate the feasibility and reproducibility of measuring a PFR and PIMR using a TPSG. Ultimately, we aimed to, for the first time, study the effect of progressive, experimentally induced microcirculatory obstruction on these novel pulmonary indices of resistance and flow. Our results demonstrate that both PFR and PIMR can detect progressive, partial obstruction of the pulmonary microcirculation, with potential implications for the improved detection of pulmonary microvascular disease.

## Methods

(See a more detailed description of pre-, intra- and post-procedural animal care in Methods S1 and its accompanying Figure S1):

### Ethics Statement

The study was conducted in accordance with the Australian Code of Practice for the Care and Use of Animals for Scientific Purposes (7th Edition, 2004) and the National Health and Medical Research Council Policy on the Care and Use of Non-Human Primates for Scientific Purposes. The study was approved by the Sydney South Western Area Health Service Animal Ethics Committee (Eastern Zone). All aspects of animal care prior to, during and following the study procedures outlined below were overseen by the veterinary team of the Australian National Baboon Colony. Any untoward events were monitored for and managed using established animal welfare and behavior protocols. Two procedures were abandoned prematurely, one due to *in situ* thrombus formation within the distal segmental pulmonary artery being studied and the other due to limited radio-contrast agent extravasation within the lung. All animals returned to normal function within 24 hours, with minimal signs of distress or discomfort. Finally, in keeping with the ethical principles of Replacement, Reduction and Refinement with relation to the use of non-human primates in medical research, experiments were only repeated until statistically meaningful results were observed. Thus, different groups of animals were utilized for different aspects of the below-described research protocol.

### Animal Model and Preparation

**Figure 1:**
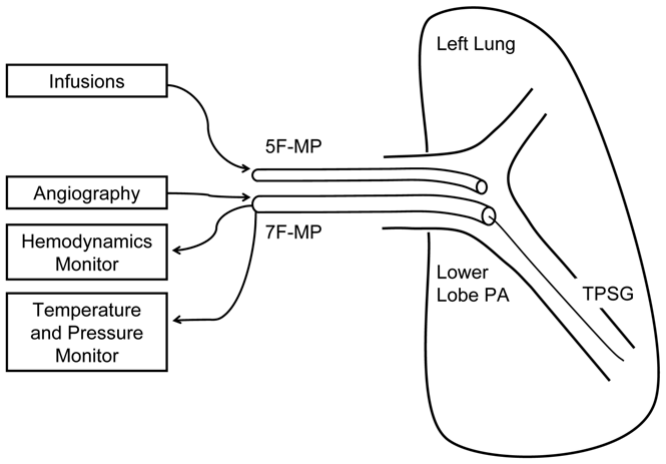
Experimental setup: Using femoral vascular access, a 7F multipurpose (MP) guiding catheter was placed in a left lower lobe segmental pulmonary artery (PA). A 5F-MP was placed alongside the 7F-MP and positioned proximal to it. A temperature and pressure sensor guidewire (TPSG) was passed through the 7F-MP and placed within the distal PA.

Thirteen healthy baboons (*Papio hamadryas*; weight 19.9±1.3 kg) were chosen for the species' anatomic and hemodynamic similarity to humans. We used a modification of our previously described methods for invasive pulmonary hemodynamic assessments in higher primates[17], as seen in Figure 1. In brief, following ketamine anesthesia, bilateral femoral venous access was established. A 7F multipurpose (MP) 55 cm guiding catheter was placed at the ostium of a left lower lobe segmental pulmonary artery and a 5F 100 cm MP guide positioned just proximal. Femoral arterial access allowed for continuous systemic hemodynamic monitoring. Following heparin (50–100 U/kg) administration, a 0.014″ TPSG (PressureWire-5 or PressureWire-Certus, Radi Medical Systems, Uppsala, Sweden) was inserted via the 7F-MP, advanced to the distal third of the segmental pulmonary artery of interest and connected to its dedicated monitoring console (Radi Analyzer-X, Radi Medical Systems, Uppsala, Sweden). Finally, we note that all aspects of animal care were delivered by a dedicated veterinary team and all animals recovered uneventfully.

### Control Measurements

Constant intravascular temperature is a prerequisite for thermodilution-derived assessments of flow. We thus recorded distal, mid and proximal segmental pulmonary artery temperatures in triplicates, using a TPSG pullback maneuver, after calibrating the TPSG to an arbitrary zero reference point within the left main pulmonary artery (n = 4).

### TPSG-Derived Mean Transit Time for the Assessment of Flow

The TPSG utilized in our study consists of a distal temperature and pressure sensor mounted on an electrically conductive shaft capable of acting as a proximal second thermistor. Fundamentally, its use for measuring flow is based on the indicator-dilution theory relating flow (*Q*), intravascular volume sampled (*V*) and mean transit-time (T*_mn_*) required for blood to traverse the sample space[10]:
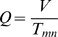
(Equation 1)


The use of TPSG for T*_mn_*-based assessment of flow was initially described in an *in vitro* system, validated against absolute flow in the canine coronary circulation[10] and then validated in the human coronary circulation[18]. In examining the utility of T*_mn_* in the primate pulmonary circulation, we have therefore carefully adapted methods originally described above, in accordance with the literature and with our previous pulmonary vascular physiology studies in high primates[17]. In particular, TPSG-derived T*_mn_* was defined (and automatically computed by the RadiAnalyzer-X console; Radi Medical Systems, Uppsala, Sweden) by expressing the distally-sensed thermodilution curve inscribed by room-temperature saline bolus-injection as a function of the time elapsed from the saline's arrival at the wire's proximal thermistor, using the following equation: 
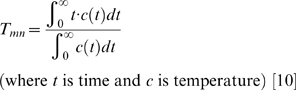
(Equation 2)


Thermodilution-derived mean transit-time assessments have been shown to be independent of indicator volume and temperature, provided that the amount utilized does not in itself alter underlying flow and adequate indicator-blood mixing occurs[10]. Thus, 3 mL room temperature saline boluses were deemed optimal for T*_mn_*-based assessment of coronary blood flow using a 6F catheter[10]. Given that indicator boluses in our study were to be administered via a larger 7F multipurpose catheter (internal volume 3 mL) and that our previous baboon work demonstrated no change in segmental pulmonary artery flow with saline boluses up to 10 mL in volume, we chose a 3.5 mL bolus for T*_mn_*-based assessment of pulmonary blood flow. Thorough flushing of the catheter with room temperature saline prior to each triplicate T*_mn_* recordings ensured that subsequent indicator boluses were of even temperature[19] allowing for use of minimum bolus volume and promoting adequate indicator-blood mixing. As thermodilution calculations are automatically gated to the indicator's arrival at the proximal TPSG thermistor (defined as a rate of temperature change greater than −3.3°C per second at this sensor) the time required to fill the catheter with saline can, by definition, be ignored. Of interest, the distal temperature sensor must detect a magnitude of change greater than 1°C in order to calculate T*_mn_*.

The distance between the tip of the 7F-MP and the distal TPSG tip was kept >6 cm at all times, in keeping with previous validation work which demonstrated that distances >6 cm were associated with significantly less T*_mn_* variability than distances <5 cm[10], likely due to improved indicator-blood mixing.

Finally, in keeping with work in the coronary circulation[10] and in order to quantify the variability of T*_mn_* measurements in the pulmonary circulation, we recorded T*_mn_* in triplicate for each hemodynamic loading condition in each animal.

### Defining Maximal Pulmonary Hyperemia

Attainment of maximal pulmonary hyperemia is fundamental to reproducible PFR and PIMR assessments. The following vasodilators were administered directly into the segmental pulmonary artery of 4 animals, in order of increasing duration of action: 1) adenosine (1 mg/mL) infusions at 10, 100, 200 and 400 µg/kg/min, for 2–3 mins each, and; 2) papaverine (3 mg/mL) boluses of 3, 6, 12, 24 and 48 mg every 2–4 mins. These agents were chosen as they have been previously demonstrated to elicit microvascular hyperemia in the pulmonary[17] and coronary beds[20], [21]. Systemic arterial pressures (as recorded by the femoral arterial pressure monitor), pulmonary arterial pressures (as sensed at the tip of the 7F-MP and recorded as peak mean PAP during a respiratory cycle, thus coinciding with expiration)[22], heart rate (as recorded from the continuous electrocardiography or systemic arterial pressure monitor) and T*_mn_* were obtained at baseline and at maximal steady-state (90–120 secs into infusions, 30–90 secs after boluses).

### Thermodilution-Derived Measurements of PFR Using TPSG

Thermodilution-derived assessment of blood flow are based upon the indicator-dilution theory relating flow (*Q*), intravascular volume sampled (*V*) and mean transit-time (T*_mn_*) required for blood to traverse the sample space[10], as noted in Equation 1.

By definition,
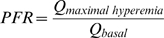
(Equation 3)


Therefore:
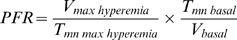
(Equation 4)


As maximal adenosine- and papaverine-induced pulmonary hyperemia occurs in the absence of change in conduit vessel diameter in the baboon[17], *V_max hyperemia_*  =  *V_basal_*. Thus, using either agent to induce hyperemia, thermodilution-derived PFR (PFR*_thermo_*) can be expressed as:
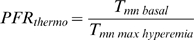
(Equation 5)


Using TPSG to measure T*_mn_* and either adenosine 200 µg/kg/min or papaverine 24 mg to induce maximal pulmonary hyperemia[17], we thus measured PFR*_thermo_* in the intact pulmonary microvasculature of 11 baboons.

### Thermodilution-Derived Measurements of PIMR Using TPSG

The relationship between flow, pressure (*P*) and resistance (*R*), obeys Ohm's law:
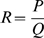
(Equation 6)


Expressing resistance in terms of *V* and *T_mn_*:
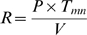
(Equation 7)


A TPSG can provide simultaneous pressure and thermodilution-derived T*_mn_* recordings. While exact resistance calculations require knowledge of arterial volume, resistance estimates can be made using a TPSG only, by calculating a resistance index (*RI*):
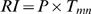
(Equation 8)


Measurement of the resistance index during *maximum microcirculatory hyperemia* (when, in theory, all available microvessels have been recruited) provides an estimate of *minimum achievable microvascular resistance*. Moreover, by recording pressure and T*_mn_* distally, the potential effect a hemodynamically significant proximal artery stenosis may have on this estimate can be mitigated. Thus, the pulmonary index of microvascular resistance (PIMR) is defined as follows:
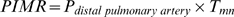
(Equation 9)


Using TPSG to measure T*_mn_* and the maximum hyperemic doses of either adenosine or papaverine to induce maximal pulmonary hyperemia, we thus measured PIMR in the intact pulmonary microvasculature of 10 animals.

### Effect of Cumulative Microvascular Obstruction on PFR*_thermo_* and PIMR

After establishing the feasibility of measuring PFR*_thermo_* and PIMR measurements using TPSG, increasing amounts of ceramic microspheres (10^4^, 10^5^ and 10^6^ particles; diameter 40–120 µm; Embospheres, Biosphere Medical, Rockland, MA) were administered into the segmental artery of 7 baboons. Microsphere sizes were chosen to obstruct the same caliber vessels as affected by human PVD (<100 µm)[2], noting that >99.8% of 50 µm diameter microspheres lodge in the baboon pulmonary circulation[23]. Baseline and maximal adenosine-induced hyperemic hemodynamics were recorded 60secs after each microsphere bolus.

### Statistical Analysis

All data are presented as mean±SEM. Analysis was performed using either Prism 4 software (GraphPad Software, San Diego, California, USA) or the SAS System for Windows version 9 (SAS Institute, Cary, North Carolina, USA). Two-way ANOVA with repeated measures was used for comparisons (unless otherwise stated) with 2-tailed p-values <0.05 regarded significant. Variability within a set of measures was calculated using the coefficient of variability. For microsphere experiments, linear mixed models were fitted to test for trend in dose response thus allowing for differing number of observations made between animals and for repeated measures within each animal.

## Results

### Effect of Sensor Position on Intravascular Temperature

The measurement of flow using indicator thermodilution method assumes a constant basal temperature within the vessel studied, attributing any change in temperature to the indicator *per se*. This assumption remains unproven in the pulmonary circulation, where blood vessels are in close proximity to air. Using a TPSG pullback maneuver, we found no temperature gradient across the segmental pulmonary artery interrogated (0.010±0.009, 0.005±0.015 and 0.010±0.009°C for distal, mid and proximal segmental pulmonary artery positions, p = 0.1).

Therefore, T*_mn_* can be used as a surrogate marker of pulmonary blood flow in the higher primate model.

### Effect of Adenosine on T*_mn_*, Hemodynamics and Cardiac Rhythm

**Figure 2:**
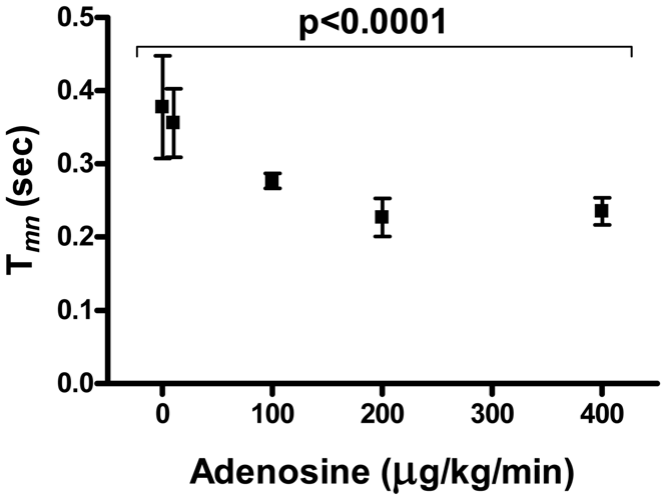
Adenosine induces dose-dependent reductions in T*_mn_*, plateauing at ≥200 µg/kg/min: T*_mn_* – thermodilution-derived mean transit time; error bars represent SEM.

Adenosine has an established role as coronary hyperemic agent[20] and was recently shown to induce pulmonary hyperemia for Doppler sensor guidewire-based PFR assessments in higher primates[17]. Increasing adenosine infusion rates produced significant, dose-dependent reductions in T*_mn_* (0.38±0.07, 0.36±0.05, 0.28±0.01, 0.23±0.03 and 0.24±0.02sec for baseline, 10, 100, 200 and 400 µg/kg/min, p<0.0001; maximal effect at 200 µg/kg/min equivalent to 40±7% reduction in T*_mn_ v* baseline; Figure 2). Concurrently, adenosine induced systemic hypotension (107±8, 102±10, 100±10, 84±5 and 78±4 mmHg for baseline, 100, 200 and 400 µg/kg/min, p = 0.0009), without affecting heart rate (93±6, 92±9, 95±8, 97±6 and 98±4 beats-per-minute for baseline, 10, 100, 200 and 400 µg/kg/min, p = 0.4) or mean PAP (12±1, 10±1, 13±4 and 16±3 mmHg for baseline, 100, 200 and 400 µg/kg/min, p = 0.3). These findings are consistent with published adenosine pharmacodynamics[17], [20].

### Effect of Papaverine on T*_mn_*, Hemodynamics and Cardiac Rhythm

**Figure 3:**
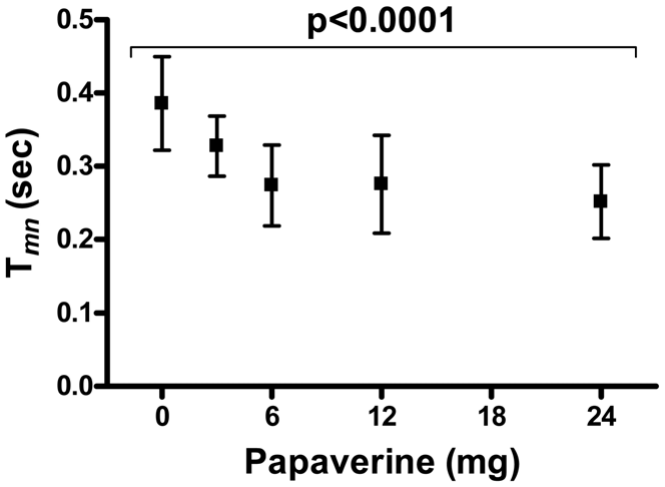
Papaverine induces dose-dependent increases in T*_mn_*: T*_mn_* – thermodilution-derived mean transit time; error bars represent SEM.

Papaverine is a known coronary hyperemic agent[21] and was recently shown to safely effect pulmonary hyperemia in higher primates[17]. Increasing papaverine boluses induced dose-dependent reductions in T*_mn_* (0.39±0.06, 0.33±0.04, 0.27±0.06, 0.28±0.07 and 0.25±0.05 sec for baseline, 3, 6, 12 and 24 mg, p<0.0001; maximal T*_mn_* reductions of 35±13% *v* baseline; Figure 3). Doses above 24 mg (up to 48 mg) were poorly tolerated due to rapid hemodynamic shifts, resulting in disruption of experimental setup. In addition to its effect on T*_mn_*, papaverine significantly increased heart rate (89±8, 90±6, 92±7, 94±8 and 104±6 beats-per-minute for baseline, 3, 6, 12 and 24 mg, p<0.0001) without affecting mean systemic pressure (106±9, 105±12, 107±11, 98±12 and 104±12 mmHg for baseline, 3, 6, 12 and 24 mg, p = 0.4) or mean PAP (19±1, 19±2, 19±2, 20±3 and 20±3 mmHg for baseline, 3, 6, 12 and 24 mg, p = 1.0). These hemodynamic effects are consistent with previously published data for papaverine[17], [24].

### Variability of T*_mn_* Measurements

The variability of T*_mn_* measurements was 10.5±1.8% and 15.6±2.7% at baseline and during maximal hyperemia, respectively.

### PFR*_thermo_* in Healthy Primates

PFR*_thermo_* values in the intact pulmonary microvasculature were calculated (Equation 5) to be 1.55±0.12 and 1.54±0.12 using adenosine and papaverine, respectively (p = 0.8 using paired t-test for complementing data sets, comparing both agents).

### PIMR in Healthy Primates

To study the effect of pulmonary hyperemia on pulmonary microvascular resistance estimates, we compared baseline, distally-measured resistance indices (RI*_basal_*) (Equation 8) with PIMR (measured at hyperemia; Equation 9). Overall, there was a statistically significant drop in microvascular resistance at maximal hyperemia (7.8±0.7 vs 5.4±0.4 mmHg.sec for RI*_basal_* vs PIMR, p<0.0001, two-tailed paired t-test). More specifically, the reduction in resistance estimates was similar for adenosine-induced hyperemia (7.8±1 vs 5.4±0.6 mmHg.sec, RI*_basal_* vs PIMR, p = 0.003, two-tailed paired t-test) and papaverine-induced hyperemia (7.7±0.6 vs 5.4±0.6 mmHg.sec, RI*_basal_* vs papaverine-derived PIMR, p = 0.006, two-tailed paired t-test).

### Effect of Progressive Microcirculatory Obstruction on Thermodilution Derived Pulmonary Hemodynamic Indices

To study the effect of progressive microcirculatory obstruction on PFR*_thermo_* and PIMR, increasing numbers of microspheres were administered into the segmental pulmonary artery. Index calculations were repeated after each bolus, using adenosine to induce hyperemia.

**Figure 4:**
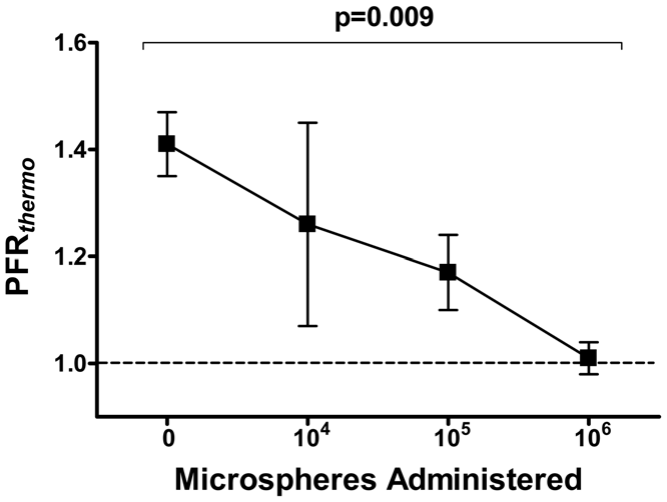
Cumulative microvascular obstruction induces progressive reductions in PFR*_thermo_*: PFR*_thermo_* – thermodilution-derived pulmonary flow reserve. The theoretical minimum value of PFR = 1 is noted by a dashed line as reference; error bars represent SEM.

Cumulative microsphere administration progressively reduced PFR*_thermo_* (1.41±0.06, 1.26±0.19, 1.17±0.07 and 1.01±0.03 for baseline, 10^4^, 10^5^ and 10^6^ microspheres bolus, p = 0.009, Figure 4).

**Figure 5:**
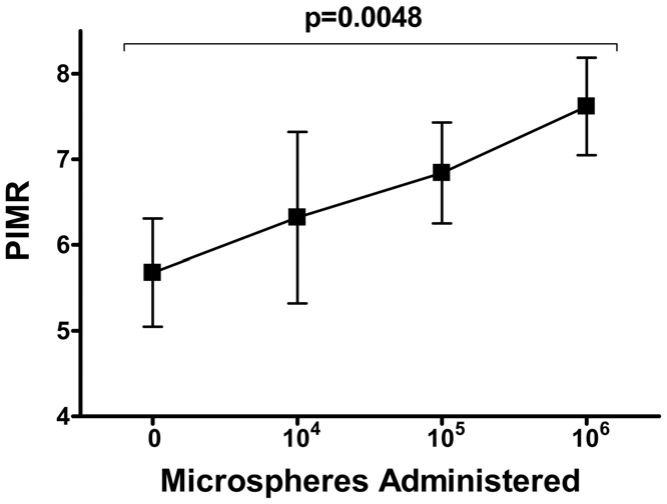
PIMR tracks progressive pulmonary microvascular obstruction: PIMR – pulmonary index of microcirculatory resistance; error bars represent SEM.

Additionally, progressive microvascular obstruction using microspheres led to a progressive rise in PIMR (5.7±0.6, 6.3±1.0, 6.8±0.6 and 7.6±0.6 mmHg.sec for baseline, 10^4^, 10^5^ and 10^6^ microspheres bolus, p = 0.0048, Figure 5). These changes occurred in the absence of microsphere effect on resting heart rate (96±5, 96±6, 95±5 and 96±6 beats-per-minute for baseline, 10^4^, 10^5^ and 10^6^ microspheres bolus, p = 0.14), mean systemic artery pressure (110±4, 116±4, 106±6 and 107±6 mmHg, p = 0.12) or mean distal segmental PAP both at maximal hyperemia (23±2, 22±2, 22±2, 21±2 mmHg, p = 0.43) and at baseline (20±2, 22±1, 21±2 and 20±2 mmHg, p = 0.85).

## Discussion

The salient findings of our study in high primates are: 1) that no significant intravascular temperature gradient exists along segmental pulmonary artery, permitting the use of thermodilution-based assessment of mean transit time for pulmonary blood flow evaluation in high primates; 2) that reproducible thermodilution-derived pulmonary flow reserve and pulmonary index of microcirculatory resistance assessments can be performed in the pulmonary circuit using either intrapulmonary adenosine or papaverine as hyperemic agents and; 3) that progressive pulmonary microvascular obstruction, of an extent insufficient to affect systemic hemodynamics or pulmonary artery pressure, results in progressive reductions in pulmonary flow reserve and elevations in pulmonary index of microcirculatory resistance. These findings suggest that both pulmonary flow reserve and pulmonary index of microcirculatory resistance may have clinical utility for improving detection of pulmonary microvascular disease.

There is increasing evidence implicating pulmonary microcirculatory dysfunction as the key pathogenetic process underlying pulmonary vascular disease[2] and chronic thromboembolic pulmonary hypertension[3]. These insidious conditions tend to present late, manifesting clinically only when the pulmonary artery pressure has risen[4] and thus portending a poor prognosis[5], [6]. The inherent insensitivity of current pressure-based diagnostic techniques for early microvascular loss and the promise of improved prognosis with early intervention[25] both provide an emergent clinical impetus for improved recognition of pulmonary microvascular dysfunction.

### Measuring Pulmonary Flow and Defining Maximal Pulmonary Hyperemia Using Thermodilution

We have recently demonstrated that PFR can be measured using a Doppler-flow velocity sensing guidewire in a higher primate model, using either adenosine 200 µg/kg/min or papaverine 24 mg to induce maximal hyperemia[17]. While Doppler-sensor guidewires measure flow velocity only[9], TPSG technology offers simultaneous assessment of pressure and thermodilution-derived flow[10], thus facilitating more comprehensive hemodynamic evaluations. Although utilized extensively in the coronary circulation[10], [18], TPSG use has not been validated in the pulmonary circuit. We demonstrate for the first time that despite the pulmonary circulation's close proximity to air, there is no temperature gradient along the pulmonary artery, supporting the use of thermodilution as a surrogate marker of pulmonary blood flow in this model for the purpose of pulmonary hemodynamic indices derivation. We demonstrate that reproducible T*_mn_* measurements can be made in the pulmonary circulation of higher primates but note that our reported variability between triplicate T*_mn_*, measures, however, was somewhat greater than the variability of Doppler flow velocity noted in our Doppler-derived PFR study[17]. Respiratory, and to a lesser degree, cardiac cycle-dependent variations in pulmonary blood flow were likely major contributors to T*_mn_* variability in the current study, with the discontinuous nature of T*_mn_* recording disallowing sufficient sampling rate frequency for cycle phase-specific gating of T*_mn_* measurement (as opposed to continuous Doppler flow velocity recordings). We postulate however, that the random distribution of T*_mn_* variability makes it unlikely to invalidate our findings (which reflect the sum of numerous recordings in multiple animals) and note that studies which validated T*_mn_* against absolute coronary blood flow[10] reported a similar T*_mn_* variability to that seen in our pulmonary work.

With regards to pulmonary hyperemia, our study demonstrates that maximal adenosine induced-flow augmentations (seen as reductions in T*_mn_*) occur at infusions rate of 200 µg/kg/min with equivalent increments in flow induced by papaverine 24 mg. These findings, and the magnitudes of flow augmentation seen at hyperemia, are similar to those noted in the Doppler PFR studies[17], suggesting reasonable correlation between Doppler flow-velocity (v*_dopp_*) and T*_mn_*, and supporting the robustness of our higher primate model. Of interest, although adenosine infusions induced the expected systemic hypotension in our study, reflex tachycardia was not seen. This finding has been previously noted in healthy humans[26] yet is seemingly at odds with our previous experience with adenosine use in baboons[17] and with other reports of adenosine use in humans[20], [27], but we suspect that it is likely a statistical phenomenon relating to the small sample size rather than a true physiologic finding.

### Thermodilution-Derived PFR Assessment in Higher Primates

Assessments of flow reserve seek to quantify the magnitude of maximum recruitable vascular reserves through the ratio of maximum hyperemic to baseline blood flow. Although coronary flow reserve evaluations have been performed using either Doppler flow-velocity or thermodilution-derived mean transit-time as surrogate measures of flow, T*_mn_*-based CFR has proven to correlate better with CFR derived from absolute flow[11]. We recently demonstrated that Doppler-derived PFR can be measured in the pulmonary circulation of higher primates[17] and thus sought to assess the feasibility of T*_mn_*-based PFR assessment in a similar model, before studying the relationship between PFR*_thermo_* and progressive microvascular obstruction.

To reproducibly measure PFR using T*_mn_* (Equation 3) we ensured that baseline measurement were made only once *all* parameters returned to the basal state and performed hyperemic measurements at steady state for adenosine or at peak effect for papaverine. As adenosine- and papaverine-induced pulmonary hyperemia in the baboon does not affect conduit vessel diameter[17], PFR*_thermo_* can be calculated using T*_mn_* only, without the need for calculating intravascular volume (Equation 4). We thus demonstrated that in the baboon intact pulmonary microvasculature, both adenosine- and papaverine-derived PFR*_thermo_* is approximately 1.5, in keeping with our recently published Doppler-derived PFR measures[17].

### PIMR Assessment in Higher Primates

IMR evaluations of the coronary circulation seek to quantify microvascular integrity by estimating *minimum achievable microvascular resistance*, a related measure to maximal vascular flow reserve assessment with CFR. By incorporating simultaneous distal coronary artery pressure and flow recordings at peak hyperemia only (as measured by a single sensor guidewire), IMR allows for more specific evaluation of the coronary microcirculation than CFR[15]. Moreover, IMR assessments have been shown to correlate with true microvascular resistance[13] and with clinical outcomes after myocardial infarction[28]. Our current findings demonstrate that reproducible PIMR measurements can be made in the baboon pulmonary circulation using either adenosine or papaverine as hyperemic agents, yielding a value of approximately 5.5 mmHg.sec in the intact lung microvasculature.

### PFR*_thermo_* and PIMR Detect Microvascular Obstruction

After assessing PFR*_thermo_* and PIMR in the intact microvasculature, we studied the ability of these novel indices to detect progressive microvascular obstruction as induced by subsegmental delivery of microspheres. We chose adenosine to induce pulmonary hyperemia for these serial hemodynamic evaluations, as its infusions provided more predictable steady-states and returns to baseline when compared to papaverine boluses. We found that increasing doses of microspheres, of a size known to obstruct the baboon pulmonary microvasculature[23], resulted in progressive reduction in PFR*_thermo_* mirrored by increases in PIMR, without affecting heart rate, mean systemic artery pressure or mean PAP. These results indicate that both PFR*_thermo_* and PIMR are capable of detecting partial pulmonary microvascular obstruction.

Our study does not compare the respective sensitivity of PFR*_thermo_* and PIMR for the detection of microvascular obstruction. We expect, however, that by obviating the need for repeated baseline assessments, PIMR may prove more reproducible than PFR*_thermo_*. Moreover, as coronary IMR is a more independent of epicardial arterial stenoses than CFR[15], we suspect that PIMR may be more specific to the pulmonary microcirculation than PFR*_thermo_*.

### Potential Clinical Implications

PFR*_thermo_* and PIMR evaluations, or variations thereof, may potentially assist in the diagnostic workup of patients suspected of having conditions such as PVD or CTEPH who are mildly symptomatic, have normal PAP yet manifest an otherwise unexplained reduced gas-transfer function (a crude indicator of available pulmonary microvascular reserve). Such evaluations may identify disease either by returning a single ‘lower than expected’ PFR*_thermo_* or ‘higher than expected’ PIMR value, or by demonstrating progressive change upon serial testing. With specific reference to CTEPH, the measurement of our proposed physiologic indices may potentially provide an improved appreciation of the extent of underlying microcirculatory dysfunction driving the disease[3] when compared to the more proximal pulmonary vessel evaluation of multi-detector computer tomogram pulmonary angiography and the qualitative evaluation of the ventilation and perfusion scan. Whether our proposed methods are superior to established diagnostic techniques remains the subject of future human studies.

### Study Limitations

We acknowledge that pulmonary microvascular obstruction using microspheres is not a validated model of pulmonary microvascular disease in humans. Our use of microspheres was aimed at simulating increases in pulmonary vascular resistance, the common pathway for pathophysiologic mechanisms underlying conditions such as PVD[29]. In keeping with this proof-of-concept study design, we also restricted our choice of hyperemic stimuli to those most commonly employed for *in vivo* assessment of the microcirculation, namely, adenosine and papaverine. However, our study cannot determine the relative contribution that either arterial or venous dilatation has made on hyperemia-induced increases in pulmonary blood flow. Nevertheless, previous work by Bhattacharya *et al* [1] in the pulmonary circulation of anesthetised dogs demonstrated that: 1) there was no measureable resistance proximal to the 50 µm arterioles or distal to the 20 µm venules and; 2) that 61% of total pulmonary vascular resistance lies proximal to the mid capillary point. These findings, along with our previous observations that maximal pulmonary hyperemic doses of both adenosine and papaverine do not affect the diameter of the segmental pulmonary arteries of baboons[17], support the notion that the proximal pulmonary microvasculature (arterioles to capillaries) is the major site of action of these hyperemic agents in the pulmonary circulation.

As is inherent in the inverse-square relationship between PAP and microvascular cross sectional area, *limited* microvascular obstruction experiments in a single lung segment will not increase resting PAP. Moreover, as the rest of the pulmonary circuit in our model was left intact, local resistance increases in the obstructed segment would have shunted blood flow to unaffected lung and its reserves, maintaining steady resting pressure throughout. Our experiments therefore cannot compare the sensitivities of our pulmonary hemodynamic indices versus PAP with regards to detection of microvascular obstruction as ethical considerations precluded microsphere administration bilaterally to the entire pulmonary circulation, leaving this question to be answered in future studies of human pulmonary microvascular disease. Interestingly, however, while microspheres reduced PFR and increased PIMR, mean PAP at peak hyperemia (where, in theory, no further blood redistribution can occur) did not change, supporting the possibility that these measures may be more sensitive than PAP for detecting microvascular obstruction.

Due to the complexity and length of the study protocol we were unable to measure cardiac output (CO) or pulmonary capillary wedge pressure (PCWP) and explore the effect of pharmacologically-induced hyperemia on CO and PCWP and the potential ‘flow-on’ effect on PFR and PIMR. Regarding hyperemia and CO, we acknowledge that the decrements in T*_mn_* seen during adenosine-induced hyperemia may, in part, be due to the drug's known ability to augment CO[26]. However, the PFR reductions and PIMR increases observed in the microsphere experiments are likely due to changes in the microvasculature rather than to reductions in cardiac output as: 1) they occurred following *partial obstruction* of a small portion of the pulmonary microvasculature (flow persisted even following maximal microsphere dose) and; 2) the microspheres did not affect heart rate, systemic or pulmonary artery pressure. These observations support our hypothesis that these indices detect variations in hyperemia-recruited microvascular reserves, though more definitive proof is required from subsequent human studies. With regards to potential hyperemia-induced changes in PCWP and their effect on physiologic index derivation, we recognize that such changes may proportionally have a greater effect on the transpulmonary pressure gradient than changes in systemic venous pressures would have on the gradient across the higher-pressured systemic circulation. Nevertheless, our current work demonstrates a relationship between PFR or PIMR and progressive microcirculatory disruption. This observation is further strengthened by an apparent inverse relationship between PIMR and PFR *per se* during microsphere administration.

The magnitude of maximal hyperemia in our model was only modest, inducing 50% augmentation in flow, equal to a PFR of 1.5. Given that pulmonary blood flow represents the entire cardiac output (CO), PFR, in theory, should be equal to the ratio of maximum attainable to baseline cardiac outputs in the animals studied. Moreover, as humans can increase CO during exercise at least 4-fold[30], we expect the normal high primate PFR to be of similar magnitude. Despite these considerations, the thermodilution-derived PFR values observed are consistent with our previously reported studies of Doppler-derived PFR in higher primates[17]. These lower-than-expected values may be species specific but are more likely a function of ketamine's known ability to depress cardiac output in vasodilated animals[31] while opposing nitric oxide dependent vasodilatation[32] (and thus limiting maximum vessel recruitment). However, even within the confines of this narrowed response range, PFR*_thermo_* was capable of detecting subtle degrees of microvascular obstruction, supporting our primary study hypothesis.

On a related note, due to technical constraints relating to obtaining stable and complete experimental setup in the smaller upper lobes of the baboons, our studies were confined to the left lower lobes of all animals. With flow in the lung traditionally divided into West zones on account of gravity-dependent variations in arterial opening pressures[33], it is possible that PFR may be different for the upper and lower lobes. However, with gravity shown to account for only 7% of pulmonary blood flow heterogeneity in the supine primate[34], PFR may potentially be less lobe-dependent in our healthy baboon model. This issue remains to be resolved in future studies, especially in the setting of pulmonary vascular disease where marked interlobar variability of pulmonary artery remodeling is seen[35].

Finally, due to the discontinuous nature of T*_mn_* recordings we could not account for the potential effect of cardiac and respiratory cycle-dependent variations in vessel diameter and flow on our T*_mn_*-derived pulmonary physiologic indices. The baboons' relatively fast heart rates and uncontrolled, spontaneous breathing further precluded such assessments. That said, we presume that the rapid, cardiac cycle-dependent undulations in flow would result in random T*_mn_* measurement error and thus have less influence on our observations as a whole. The fact that T*_mn_* has been shown to correlate strongly with absolute blood flow in the coronary circulation[10], where the cardiac cycle would presumably have a similar effect on vessel diameter, further supports this argument. With regards to the known effect of respiration on hemodynamics, we mitigated mean PAP sampling error by only recording peak mean PAP during each respiratory cycle (occurring at peak expiration in the spontaneously breathing animal)[22]. We propose, in fact, that respiratory-dependent changes in T*_mn_* likely contributed, at least in part, to the larger variability of triplicate T*_mn_* recordings (10.5%) when compared to that of continuously-sampled, respiratory cycle-gated Doppler flow velocity (3.3%) in the baboon's pulmonary circulation (3.3%)[17]. The similarity between thermodilution and Doppler derived PFR however suggests that overall, the effect of respiration on T*_mn_*-derived flow indices may be lessened by repeated sampling.

### Conclusion

Diagnostic techniques for the detection of pulmonary microvascular abnormalities are lacking. We demonstrate that safe and reproducible assessment pulmonary flow reserve and pulmonary index of microcirculatory resistance can be performed in a primate model, using a temperature and pressure sensor guidewire and either adenosine or papaverine as hyperemic agents. Moreover, we show that these indices track progressive pulmonary microvascular obstruction. Although suggestive of potential clinical use, further studies are required to validate and evaluate the utility of these novel indices of pulmonary microvascular status in humans.

## Supporting Information

Methods S1 Online methods supplement
(0.05 MB DOC)

Figure S1 Experimental setup: Using femoral vascular access, a 7F multipurpose (MP) guiding catheter was placed in a left lower lobe segmental pulmonary artery (PA). A 5F-MP was placed alongside the 7F-MP and positioned proximal to it. A temperature and pressure sensor guidewire (TPSG) was passed through the 7F-MP and placed within the distal PA.
(0.16 MB TIF)
