# Supplementary material for: Measurement of Pulmonary Flow Reserve and Pulmonary Index of Microcirculatory Resistance for Detection of Pulmonary Microvascular Obstruction
Source: PLoS One. 2010 Mar 9;5(3):e9601. doi: 10.1371/journal.pone.0009601 (PMC2834756; doi:10.1371/journal.pone.0009601)
Supplement: Methods S1 — Online methods supplement (0.05 MB DOC) [file pone.0009601.s001.doc]

**ONLINE METHODS SUPPLEMENT**

**“Measurement of Pulmonary Flow Reserve and Pulmonary Index of Microcirculatory Resistance for Detection of Pulmonary Microvascular Obstruction”**

**Ethics Statement**

The study was conducted in accordance with the Australian Code of Practice for the Care and Use of Animals for Scientific Purposes (7th Edition, 2004) and the National Health and Medical Research Council Policy on the Care and Use of Non-Human Primates for Scientific Purposes. The study was approved by the Sydney South Western Area Health Service Animal Ethics Committee (Eastern Zone). All aspects of animal care prior to, during and following the study procedures outlined below were overseen by the veterinary team of the Australian National Baboon Colony. Any untoward events were monitored for and managed using established animal welfare and behavior protocols. Two procedures were abandoned prematurely, one due to in situ thrombus formation within the distal segmental pulmonary artery being studied and the other due to limited radio-contrast agent extravasation within the lung. All animals returned to normal function within 24 hours, with minimal signs of distress or discomfort. Finally, in keeping with the ethical principles of Replacement, Reduction and Refinement with relation to the use of non-human primates in medical research, experiments were only repeated until statistically meaningful results were observed. Thus, different groups of animals were utilized for different aspects of the below-described research protocol.

**Animal model and preparation**

Healthy baboons (*Papio hamadryas*; 3 female, 10 males; weight 19.9±1.3kg) were chosen for the species’ anatomic and hemodynamic similarity to humans. The study was approved by our institutional review board and all aspects of animal preparation, procedures and recovery were overseen by a veterinarian from our institution. We used a modification of our previously described methods for invasive pulmonary hemodynamic assessments in higher primates[1], as seen in Figure S1. To recapitulate, each study animal was fasted the night before the procedure and transported along with a companion to the local animal holding facility, in order to reduce anxiety. General anesthesia was induced using intramuscular ketamine (5-8mg/kg), clonazepam (0.01mg/kg; as prophylaxis for ketamine-induced seizures) and atropine (0.04mg/kg; to minimize airways secretions). Intravenous ketamine infusions (0.2mg/kg/min) maintained anesthesia with all animals breathing spontaneously throughout. Parenteral carprofen (3mg/kg; non steroidal analgesic) and metoclopramide (5mg; dopamine antagonist antiemetic) were administered peri-procedurally at the discretion of the animal carer. An indwelling urinary catheter was inserted to prevent bladder distention an assist in fluid management. The anesthetized animals were then placed supine on the angiography suite’s examination table (Axiom Artis; Siemens, Munich, Germany), fitted with Hudson mask delivering supplemental oxygen, connected to electrocardiographic monitoring and had both groins shaved, sterilized and draped. Bilateral femoral venous access was established, using aseptic Seldinger technique and fluoroscopic guidance. Via a femoral vein, a 7F multipurpose (7F-MP) 55cm guiding catheter was placed at the ostium of a left lower lobe segmental pulmonary artery. A 5F 100cm multipurpose guide was inserted via the opposite femoral vein and placed in the distal left main pulmonary artery, just proximal to the 7F-MP, enabling direct intra-pulmonary artery drug infusions. A 4F femoral arterial line facilitated continuous systemic blood pressure monitoring. Following heparin (50-100U/kg) administration, a 0.014” temperature and pressure sensor guidewire (TPSG; PressureWire-5 or PressureWire-Certus, Radi Medical Systems, Uppsala, Sweden) was inserted via the 7F-MP and its tip advanced at least 6 cm past the guide[2], into the distal third of the segmental pulmonary artery of interest. Iopamidol (Isovue 370; Regional Health Care Products Group, Sydney, NSW, Australia) was used as radio-contrast agent. The TPSG was then connected to its console (RadiAnalyzer X, Radi Medical Systems, Uppsala, Sweden).

Experimental microcirculatory disruption was undertaken using increasing amounts of ceramic microspheres (10^4^, 10^5^ and 10^6^ particles; diameter 40-120µm; Embospheres, Biosphere Medical, Rockland, MA) administered into the segmental artery of 7 baboons. Microsphere sizes were chosen to obstruct the same caliber vessels as affected by human PVD (<100µm)[3], noting that >99.8% of 50µm diameter microspheres lodge in the baboon pulmonary circulation[4]. Of note, the use of microspheres did not affect post procedural recovery.

**Post Procedural Animal Care**

At the end of the procedure, the anesthetized animal was transferred back to the local animal holding facility where sedation was ceased. The femoral vascular access sheaths were removed and manual pressure applied continuously to both groins for one hour in order to prevent local groin complications. The peripheral cannulae and indwelling urinary catheter were then removed, and any vascular puncture site dressed using compressive bandage. The recovering yet still drowsy animal were then transferred to their holding cage and placed in the left decubitus recovery position, in an attempt to protect the airway in case of emesis. Once awake and free of sedative induced ataxia, the animals were fed and transported back to the National Baboon Colony along with their companion.

**References:**

1. Ilsar R, Chawantanpipat C, Chan KH, Waugh R, Hennessy A, et al. (2009) Measurement of pulmonary flow reserve in higher primates. Clinical and Experimental Pharmacology and Physiology 36: 797-802.

2. De Bruyne B, Pijls NH, Smith L, Wievegg M, Heyndrickx GR (2001) Coronary thermodilution to assess flow reserve: experimental validation. Circulation 104: 2003-2006.

3. Pietra GG, Capron F, Stewart S, Leone O, Humbert M, et al. (2004) Pathologic assessment of vasculopathies in pulmonary hypertension. Journal of the American College of Cardiology 43: 25S-32S.

4. Lovering AT, Stickland MK, Kelso AJ, Eldridge MW (2007) Direct demonstration of 25- and 50-microm arteriovenous pathways in healthy human and baboon lungs. American Journal of Physiology - Heart & Circulatory Physiology 292: H1777-1781.

**Supplemental Figure Legend**

**Figure S1. Experimental setup:** Using femoral vascular access, a 7F multipurpose (MP) guiding catheter was placed in a left lower lobe segmental pulmonary artery (PA). A 5F-MP was placed alongside the 7F-MP and positioned proximal to it. A temperature and pressure sensor guidewire (TPSG) was passed through the 7F-MP and placed within the distal PA
