# Supplementary figures and images for: Measurement of Pulmonary Flow Reserve and Pulmonary Index of Microcirculatory Resistance for Detection of Pulmonary Microvascular Obstruction
Source: PLoS One. 2010 Mar 9;5(3):e9601. doi: 10.1371/journal.pone.0009601 (PMC2834756; doi:10.1371/journal.pone.0009601)

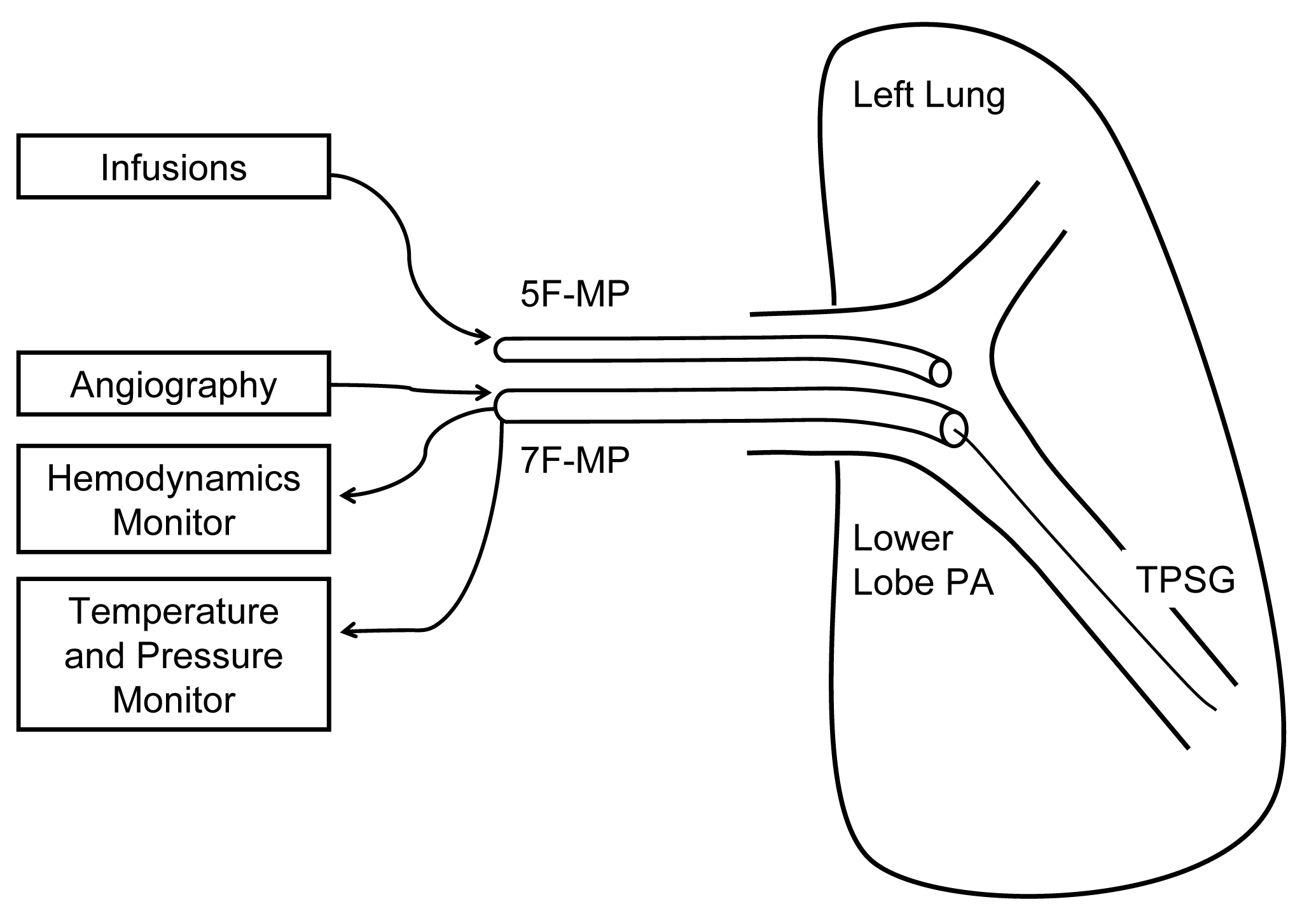

Supplement: Figure S1 — Experimental setup: Using femoral vascular access, a 7F multipurpose (MP) guiding catheter was placed in a left lower lobe segmental pulmonary artery (PA). A 5F-MP was placed alongside the 7F-MP and positioned proximal to it. A temperature and pressure sensor guidewire (TPSG) was passed through the 7F-MP and placed within the distal PA. (0.16 MB TIF) [file pone.0009601.s002.tif]
